# Mosquito Extermination and Its Bearing on the Yellow Fever and Modern Quarantines*This paper was read at the Conference of City and County Health Officers, held Austin with the State Health Department, May 2d, 1906.

**Published:** 1906-05

**Authors:** C. W. Truehart

**Affiliations:** Galveston City Health Officer; Galveston, Texas


### TEXAS MEDICAL JOURNAL.

ESTABLISHED JULY, 1885.

PUBLISHED MONTHLY.—SUBSCRIPTION $1.00 A YEAR.

VOL. XXI. AUSTIN, MAY, 1906. No. 11.

The publisher is not responsible for the views of contributors.

For Texas Medical Journal.

### Mosquito Extermination and Its Bearing on the Yellow Fever and Modern Quarantines.*

BY C. W. TRUEHART, M. D., GALVESTON CITY HEALTH OFFICER.

No truth in the realm of modern sanitary science has been more conclusively demonstrated than the fact that the female stegomyia fasciata mosquito is absolutely the only agency by which yellow fever can be spread, and that, therefore, the thorough and systematically maintained extermination of this mosquito constitutes the whole gist, the *sine qua non,* in successfully dealing with the yellow fever question; and, indeed, if rightly appreciated, this fact also puts a radically new phase on the question of modern quarantines. With this mosquito extinct, the disease is robbed of its terrors, the liability to its invasion lessened, its spread rendered certain of control, and even its ultimate and permanent banishment from Texas made possible.

Now, the question naturally presents itself, is such a complete destruction of this mosquito possible? Extended practical experience in Galveston, Havana and other places, justifies the assertion that beyond the question of a doubt such extermination is practicable. What man has done man can do. It is only a question of systematic and persistent effort on the part of health authorities cordially and efficiently supported by the people.

In Galveston, by dint of a rigid enforcement of the city ordinances requiring the effectual covering in or screening of all cisterns so as to absolutely prevent the access of mosquitoes to the

*This paper was read at the Conference of City and County Health Officers, held Austin with the State Health Department, May 2d, 1906.

contained water, the treating with crude carbolic acid of all closet and waste water sinks and all water held in barrels, buckets or other vessels for fire extinguishing purposes and keeping the surface of all standing fresh water not readily drained off, continually coated with crude petroleum oil, this species of mosquito has been practically exterminated, and, indeed, other varieties of the pestiferous insects so far gotten rid of as to greatly add to the comfort of life and the health of man and beast in this locality.

The use of crude carbolic acid in the closet sinks not only prevents the breeding of mosquitoes, but also the flies. This insect is unquestionably, in considerable part, responsible for the spread of diphtheria, scarlet fever and typhoid fever.

In San Antonio, so I am credibly informed, the same success in combating mosquitoes has been secured.

The stegomyia fasciata, let it be borne in mind, is a thoroughly domestic insect—as much a domestic insect as barnyard chickens are domestic fowls. It does not breed in marshes, but almost exclusively in proximity to human habitations, and, as a rule, only in clean, fresh water. These peculiarities render this mosquito readily accessible and comparatively easy of being dealt with. Cutting any species of mosquito off from water to drink as well as to breeding, and a resort to fumigation of the houses, will effectually rid any place of the insects.

Formaldehyde gas I have found to be the least obnoxious and by far the most effective agent as an insecticide and germicide. But the secret in securing the fullest measure of effectiveness from formaldehyde gas depends upon the generation of the gas with such rapidity and in such large volume and concentrated strength as to overwhelm and destroy insect and germ life. One hour of such fumigation suffices to kill all insects and disease germs. In the past three years, since the extermination of the stegomyia fasciata mosquitoes, time and again have cases of well-marked yellow fever been introduced into Galveston. The cases were promptly taken in hand, the ship or rooms which had been occupied by them, and also adjacent premises, thoroughly fumigated, the patients conveyed in our screened ambulance right through the heart of the city and cared for in a screened room at hospital, the State health officer promptly wired, and the community at once informed as to the existence of the disease, the measures resorted to to control its spread and as to all facts in the matter. This, too, was in such mild weather that in olden times great alarm would have resulted and the disease been very liable to have spread. The cases were nursed by non-immune nurses and clinical classes from the medical college freely brought into the room to study the disease. I have steadily set my face against the plan of secrecy and deception that has in past years lamentably obtained in some localities. Let health officers take the public into their confidence; be always open and above-board with the people; this is the best way to allay public apprehension, prevent panics and secure the efficient co-operation of the physician and the laity in stamping out the disease in any locality.

The success achieved in the extermination of the mosquito in Havana by the United States army officers, and the wonderful exemption from yellow fever resulting therefrom as long as the extermination was maintained, are matters of indisputable official record. Dr. W. B. McLaughlin, a gentleman well known in Austin for reliability and scientific attainments, who was in the United States army service during and following the extermination of the stegomyia fasciata mosquitoes in that city by the lamented United States Army Surgeon Reed and his co-laborers, informs me of his own personal observation and knowledge, that after the extermination of the mosquitoes, vessels from the tropics with yellow fever aboard arrived at that port frequently. The passengers and crews were held at the quarantine detention station for five or six days, the fever patients carefully screened, sent ashore in a launch and conveyed in screened ambulance through the crowded streets and cared for in a screened hospital; the ship, after fumigation to kill any mosquitoes lurking in her, was allowed to at once come up to the quay, discharge cargo and load again. And all this without any spread of the disease. This is the scientific and common-sense plan upon which all quarantines in our enlightened day should be conducted.

The rich and growing commerce of Cuba and other West India islands, Mexico, Central and South America would naturally seek our Texan and other gulf ports if only our own people would wake up to the fact that with the yellow fever-spreading mosquito exterminated, this valuable trade could be handled without danger to the health and lives of our citizens. The antiquated quarantine methods that have heretofore prevailed in gulf ports have driven this commerce to their North Atlantic rivals and to Europe. Let us stop thus cutting our own throats.

The time is at hand when the light shed on the subject by modern sanitary science shall soon dispel the ignorance and superstition of the past and when these needless and now senseless quarantine restrictions to passenger and freight traffic will no longer be tolerated at the hands of either State or Federal health authorities.

As to the all-important work of mosquito extermination, it will not do to confine this to the seaports and larger cities and towns. State laws should be enacted requiring the screening, etc., of water retainers and other measures to be carried out about all human habitations all over the State.

Such widespread and effective mosquito extermination is, however, quite out of the question for marine hospital doctors to accomplish. Such task can, in the very nature of things, only be achieved by local men, city and county health officers and employes under their control, faithfully assisted by the people and under the general direction of the State Health Department.

For the sake of argument, let it for the moment be admitted that with the ignorance prevailing, and the current trend of public notions, the people being but partially educated up to the point of accepting and acting upon the teachings of the mosquito theory and all that it means,—let it be admitted that widespread and radical extermination of the mosquitoes can not at once be attained all over the State. Is not a partial measure of protection for man and beast worth striving for and far better than no protection at all? Let us physicians, as custodians of the public health, educate the people in our respective localities as to the best means for protection against yellow fever, malarial diseases, etc. Let us contend for thorough work, for the highest ideal—total extermination of the whole genus mosquito.

Galveston, Texas, May 1, 1906.

